# A Chinese Prescription Yu-Ping-Feng-San Administered in Remission Restores Bronchial Epithelial Barrier to Inhibit House Dust Mite-Induced Asthma Recurrence

**DOI:** 10.3389/fphar.2019.01698

**Published:** 2020-01-31

**Authors:** Kaifan Bao, Weiyuan Yuan, Yijing Zhou, Yanyan Chen, Xuerui Yu, Xiaoyu Wang, Zhirong Jia, Xi Yu, Xiaotong Wang, Lu Yao, Siqi Wang, Yifan Xu, Yuheng Zhang, Jie Zheng, Min Hong

**Affiliations:** ^1^ Jiangsu Key Laboratory for Pharmacology and Safety Evaluation of Chinese Materia Medica, School of Pharmacy, Nanjing University of Chinese Medicine, Nanjing, China; ^2^ School of Basic Medicine and Clinical Pharmacy, China Pharmaceutical University, Nanjing, China; ^3^ The Nanjing Han & Zaenker Cancer Institute (NHZCI), OG Pharmaceuticals, Nanjing, China

**Keywords:** asthma recurrence, Yu-Ping-Feng-San, remission, bronchial epithelial barrier, DSG1, TSLP

## Abstract

Clinically, the treatments against asthma like β_2_ agonist focus on controlling the symptoms rather than inhibiting recurrence radically. This study aims to evaluate the efficacy and mechanism of a potent Chinese prescription Yu-Ping-Feng-San (YPFS) against asthma recurrence. We here established an optimized house dust mite (HDM)-induced asthma recurrence mice model with typical asthmatic responses such as significantly augmented airway hyperresponsiveness (AHR), elevated serum IgE, pulmonary type 2 cytokines IL-5 and IL-13 levels, pathological changes including thickening bronchial wall, inflammatory infiltration of lung tissue, etc. Moreover, all typical asthmatic pathological features were prominently alleviated by YPFS applied during remission phase ahead of second elicitation, which was even more effective than three different types of medications dexamethasone, montelukast and salbutamol, which were commonly applied in clinical practice, administered during recurrence phase. Besides, we found that desmoglein 1 (DSG1) remained deficient when asthmatic responses regressed whereas tight junction (TJ) claudin 1 (CLDN1) or adherin junction (AJ) E-cadherin restored spontaneously. *In vitro*, DSG1 interference resulted in increased thymic stromal lymphopoietin (TSLP) secretion, and epithelial barrier compromise evidenced by significantly elevated transepithelial electrical resistance (TEER) and increased 4-kDa FITC-dextran influx. YPFS could downregulate TSLP production and restore HDM-induced DSG1 deficiency and barrier destruction, which was further reversed by shDSG1. Collectively, administration of YPFS in remission prominently alleviated HDM-induced asthma relapse by restoring DSG1 and decreasing TSLP overexpression, which might be the key factors contributing to chronic asthma relapse. Our data not only demonstrated the pivotal role of DSG1 in asthma pathogenesis, but also provided a novel and potent therapeutic strategy against chronic asthma.

## Introduction

Characterized by airway hyperresponsiveness and chronic inflammation, asthma has now become a severe public health problem which causes over 300 million people worldwide (Papi et al., 2018). Asthmatic patients are commonly suggested to use β_2_ agonist, inhaled corticosteroids, muscarinic antagonists, leukotriene receptor antagonists, anti-IgE, or interleukin-5 (IL-5) neutralizing antibodies, etc. depending on the severity of their conditions (Pelaia et al., 2012; Papi et al., 2018). Considering that these treatment aims to control the symptoms instead of reducing the risk of flare-ups and adverse effects are basically inevitable, it urges us to promote novel approaches with less or negligible side-effects to reduce the recurrence rate of asthma.

Under these circumstances, as a potent traditional Chinese medicine (TCM) against allergic disorders, YPFS applied in the remission of asthma has been proven efficient clinically through thousands of years of Chinese history (Chan et al., 2016). Our group previously reported that YPFS, which consists of huangqi (HQ, *Astragalus mongholicus Bunge*), baizhu (BZ, *Atractylodes macrocephala Koidz*), and fangfeng (FF, *Saposhnikovia divaricata (Turcz.) Schischk)*, ameliorated atopic dermatitis (AD) recurrence by restoring epithelial junctions and reducing alarmin cytokines IL-33 and TSLP (Zheng et al., 2018). Furthermore, by means of bioactive components screening, we found that ingredients in YPFS including astragaloside-IV, calycosin, and cimifugin possessed anti-allergic efficiency at the initial stage of AD (Bao et al., 2016; Wang et al., 2017; Jia et al., 2018). However, the activity and the underlying mechanism of YPFS against asthma recurrence remains to be investigated.

Recently, accumulated evidences suggest that epithelial barrier dysfunction play a pivotal role in the pathogenesis of type 2 inflammatory diseases including asthma, AD, eosinophil esophagitis (EoE) (Georas and Rezaee, 2014; Schleimer and Berdnikovs, 2017). Epithelial barrier is constructed mainly by TJs, AJs, and desmosomes. The compromise of TJs and AJs, represented by claudins, occludin, ZO-1, and E-cadherin, leads to the secretion of epithelial-derived pro-allergic cytokines IL-33, IL-25, and TSLP, which starts to orchestrate type 2 inflammation through type 2 T helper cells (Th2) or type 2 innate lymphoid cells (ILC2), subsequently (Loxham and Davies, 2017; Schleimer and Berdnikovs, 2017; Sugita et al., 2018). As one major component of desmosome, DSG1 has been well characterized in inflammatory skin disease named severe dermatitis, multiple allergies, and metabolic wasting (SAM) syndrome (Samuelov et al., 2013). In contrast with the well-established recognition of TJs and AJs, few evidences have unraveled the role of desmosome in asthma pathogenesis. In fact, only one study so far has reported that DSG1 might be one of the key biomarkers associated with rhinovirus-induced asthma (Liu et al, 2016). Since DSG1 has been found to be critical for epithelial barrier, and its dysfunction leads to inflammation of skin and esophagus, we speculated that the perturbation of DSG1 might also contribute to asthma pathogenesis.

In this study, we compared the efficacy of three clinically applied medications with YPFS against asthma recurrence on an optimized mice model. And by investigating the activity of YPFS regulating bronchial epithelial junctions *in vivo* and *in vitro*, we elucidated the underlying mechanism of YPFS applied in remission phase against asthma recurrence.

## Materials and Methods

### Materials

House dust mite (HDM, dermatophagoides pteronyssinus) was purchased from Greer Laboratories. Dexamethasone injection (DEX) was a product from Chenxin Pharmaceutical Co., Ltd (Jining, China). Salbutamol Sulfate Sustained Release Tablets (SAL) were purchased from YaBang AiPuSen Pharmaceutical Co., Ltd (Yancheng, China). Montelukast sodium tablets (MON) were purchased from Merck Sharp & Dohme Ltd (K004567, Hangzhou, China). Botanical identification of huangqi (HQ, *Astragalus mongholicus Bunge*), baizhu (BZ, *Atractylodes macrocephala Koidz*), and fangfeng (FF, *Saposhnikovia divaricata (Turcz.) Schischk)* purchased from Nanjing Pharmaceutical Company (Nanjing, China) was confirmed by Professor Chungen Wang (Nanjing University of Chinese Medicine). All voucher specimens (no. NZY-HM-2013001 for HQ; no. NZY-HM-2013004 for BZ; and no. NZY-HM-2013005 for FF) are deposited in the Herbarium of Traditional Chinese Medicine, Nanjing University of Chinese Medicine.

### Preparation of YPFS

Preparation of YPFS and establishment of the HPLC fingerprint of the prescription were conducted as described previously (Shen et al., 2014). Briefly, 300 g HQ, 100 g BZ, and 100 g FF were extracted twice with ethanol: water (95:5, V/V) and evaporated by a vacuum concentrator to 1.5 g crude drug/g extracts. Furthermore, the HPLC fingerprint of YPFS extracts was established as well (**Figure S1** and **Table S1**). A total of 32 well-separated peaks were identified in the fingerprint of YPFS extracts at 254 nm, and the dominating compounds were prim-O-glucosylcimifugin, calycosin-7-glucoside copyranoside, cimifugin, 4'-O-glucopyranosyl-5-O-methylvisamminol, ononin, calycosin, sec-o-glucosylhamaudol, and formononetin. Besides, the semisolid extracts of YPFS were then prepared in phosphate buffer saline (PBS) for further study. *In vivo*, 0.1 ml/10g body weight of YPFS at 433.3 mg extracts/ml, which equals to 6.5 g crude drug/kg, was administered *via* intragastric. And 100 μg/ml of YPFS was used for *in vitro* study.

### Animals

Male BALB/c mice of 6–8 weeks old were purchased from Shanghai Slac Laboratory Animal Company (Shanghai, China). All animals were maintained at Nanjing University of Chinese Medicine under specific pathogen-free conditions at 18°C–25°C and 50%–60% humidity. All procedures involving animals were approved by the Animal Care and Use Committee of Nanjing University of Chinese Medicine, and strictly performed according to the Guide for the Care and Use of Laboratory Animals.

### Experimental Mice Model and Dosage Regimens

Based on an optimized HDM-induced asthma mice model (data not shown), an HDM-induced asthma recurrence model was duplicated as follows: on day 0, 7, and 14, mice were injected intraperitoneally with 50 μg HDM (0.5 mg/ml), and intranasally elicited with 25 μg HDM (2.5 mg/ml) for three times on day 21–23. When the airway resistance decreased to the similar level until day 30, model mice were intranasally exposed to 25 μg of HDM (2.5 mg/ml) on day 38–40 for another elicitation, i.e., asthma recurrence. After being anesthetized by isoflurane, indicated samples were collected 24 h post last elicitation.

To compare the efficiency of YPFS with three commonly used medications against asthma in clinical practice, YPFS at 6.5 g crude drug/kg (converted from clinical dosage based on body surface area normalization) were administered intragastrically (i.g.) once daily during remission phase on day 31–37 while three medications were administered for 7 days during remission or 3 days during second elicitation. Specifically, 0.67 mg/kg DEX was administered intraperitoneally (i.p.) while MON at 1.3 mg/kg and SAL at 0.78 mg/kg were given i.g. once daily. The administration of YPFS referred to the data we published before (Zheng et al., 2018), which is the equivalent dosage switched from clinical application, and also the most efficient dosage against experimental AD recurrence. For all the animal experiments involved in this study, mice in control group were administered by the same volume of PBS, i.e., the solvent.

### Airway Resistance Measurement

Under general anesthesia by isoflurane, airway resistance of mice was determined to increasing doses (0, 0.0625, and 0.125 mg/kg) of methacholine utilizing the Buxco PFT Controller and the FinePointe^®^ PFT software (Data Sciences International, MN, USA) as described before (Maazi et al., 2018).

### Cell Counting

Eosinophils in blood was counted using an eosinophil direct counting kit (Jiancheng Bioengineering Institute, Nanjing, China). Total cells in bronchoalveolar lavage fluid (BALF) was counted *via* Countstar (Shanghai, China). And eosinophils, neutrophils, macrophages, and lymphocytes in BALF were microscopically enumerated after cell pellet in BALF was dyed using a Wright-Giemsa stain kit (Jiancheng Bioengineering Institute, Nanjing, China).

### Immunohistochemistry

Mice lung tissue were fixed with 10% paraformaldehyde and embedded in paraffin. Sections (6 μm) were prepared and immunostained with antibody against DSG1 (Abcam, Shanghai, China) according to manufacturer's protocols. In all cases, analysis was restricted to areas of well-orientated and structurally intact epithelium.

### Histological Assay

Lung tissue sections prepared as described in immunohistochemistry assay were stained with hematoxylin and eosin (H&E).

### Air-Liquid Interface (Ali) Culture

Human bronchial epithelial cells (16HBE) were cultured in RPMI 1640 medium with 10% FBS and 1.8 mM Ca^2+^ and differentiated for 28 days at air-liquid interface using Transwell^®^ Permeable Supports (Costar, ME, USA) after cells formed a confluent monolayer.

### shRNA Construct and Transfection

shRNA was constructed and cloned into the lentivirus vector pLenti, subsequently, by Asia-vector Biotechnology Co. LTD (Shanghai, China). The oligonucleotide sequence of the shRNA targeting DSG1 was 5'- CGTTGTTAGTGGACACCCA -3'. After 24 h in culture after seeding, shRNAs were transfected into 16HBE cells at a final concentration of 3 μg/ml with Lipofectamine 2000 (Life Technologies, CA, USA) according to the manufacturer's protocol. To further verify the key role of DSG1 for YPFS to exert the bronchial epithelial protective activity, shRNAs were transfected into 16HBE cells subsequent to pre-incubation with YPFS at 100 μg/ml for 24 h. The dosage of YPFS referred to the data which we had demonstrated as the most efficient concentration restoring skin epithelial barrier (Zheng et al., 2018).

### Measurement of Transepithelial Electrical Resistance (TEER) and Paracellular FITC-Dextran Flux

TEER and paracellular flux measurements were performed as described previously (Zheng et al., 2018). Briefly, TEER was measured using an EVOM2 (World Precision Instruments, FL, USA) daily after ALI culture from day 0 to day 28. Permeability of the stratified 16HBE cells was analyzed on day 28 using 4kDa FITC-dextran.

### Western Blot Analysis

Cell or tissue lysates was subjected to SDS-PAGE and Western blot analysis with the use of anti-claudin1 (LifeSpan BioSciences, WA, USA), anti-E-cadherin (R&D Systems, MN, USA), anti-ZO-1 and anti-occludin (Proteintech group, Wuhan, China), anti-DSG1 (Invitrogen, CA, USA), and anti-GAPDH (Bioss, Beijing, China) antibodies followed by horseradish peroxidase–electrochemiluminescence (HRP-ECL) detection (Millipore, MA, USA).

### Quantitative Real-Time PCR

Gene expressions of DSG1 and TSLP in lung tissue or 16HBE cell were detected by quantitative real-time PCR as described previously (Bao et al., 2016) with changes as follows: the oligonucleotide sequences of primers (GenScript Biotech Corp, Nanjing, China) applied were 5'-TACTATACTCTCAATCCTATCCCTG-3' (S) and 5'-ACTTCTTGTGCCATTTCCTG-3' (AS) for TSLP; 5'- TGGGGAATATAAAGGAACAGTGCT-3' (S) and 5'- CGTTGTGGGTTCTCAGTGGA (AS) for DSG1; 5'-GGTTGTCTCCTGCGACTTCA-3' (S) and 5'-TGGTCCAGGGTTTCTTACTCC-3'(AS) for GAPDH.

### Enzyme-Linked Immunosorbent Assays (ELISA)

BALF of mice was collected as described previously (Bao et al., 2018). Indicated cytokines in BALF, lung tissue homogenate and cell culture medium and IgE in serum were assessed using Ready-Set-Go! ELISA kits (eBioscience, CA, USA) according to the manufacturer's instructions. All measurements were made in triplicate.

### Statistical Analysis

Data are expressed as means ± standard deviations (SD). Comparisons between two groups were conducted by *t-*test (unpaired), and multiple groups were compared with one-way analysis of variance and two groups with Dunnett's test, using GraphPad Prism 7 (GraphPad Software, CA, USA). A statistical value of *P* < 0.05 was considered significant.

## Results

### Establishment of an Asthma Recurrence Mice Model Induced by HDM

**Figure 1:**
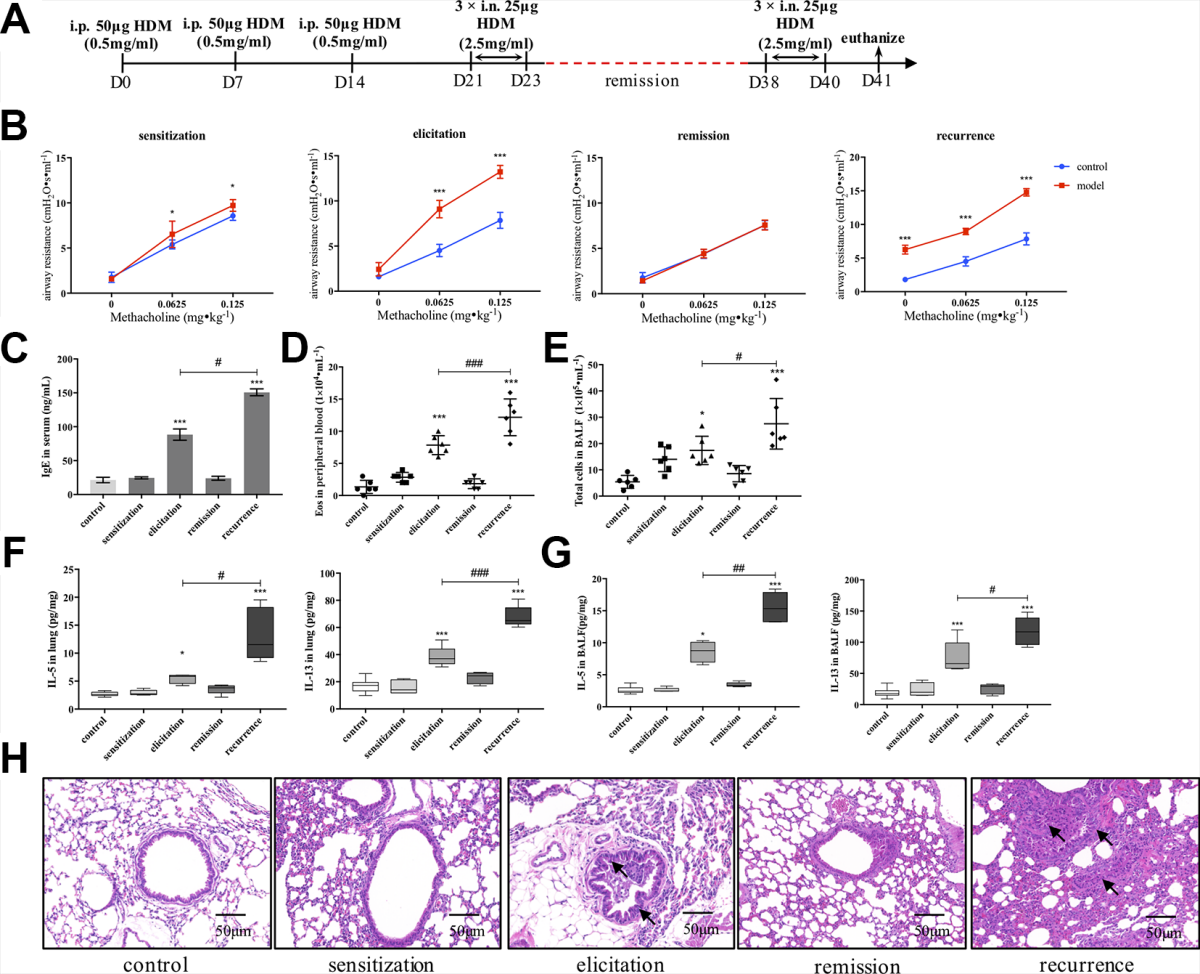
Establishment of an HDM-induced asthma recurrence model. **(A)** Flowcharts of the HDM-induced asthma recurrence model. To establish an mice recurrence model analogous to chronic asthma on human subjects, established asthmatic mice were re-elicited with HDM on days 38–40 after airway resistance recovered to normal level. **(B)** AHR of mice was evaluated by measuring airway resistance to increasing doses of methacholine on day 15 (sensitization), day 24 (elicitation), day 31 (remission), and day 41 (recurrence). At indicated time points mentioned above, **(C)** IgE in serum was detected by ELISA; **(D)** Eosinophils in peripheral blood were enumerated; **(E)** Total cells in BALF were counted; type 2 cytokines IL-5 and IL-13 in **(F)** lung homogenates and **(G)** BALF were detected. (All data represent mean ± SD from three independent experiments performed in triplicate, n = 6, ^*^ *P* < 0.05, ***P < 0.001 *versus* control; ^#^ *P* < 0.05, ^###^ *P* < 0.001 *versus* elicitation); ^##^ *P* < 0.01 *versus* elicitation **(H)** Histological changes of lung sections were determined by H&E staining (n = 7; scale bar = 50 μm). Solid black arrows indicate that bronchial wall thickened after first elicitation, and much more severe pathological changes were observed in recurrence. Collectively, re-exposure to HDM markedly augmented the type 2 inflammatory responses compared to that after first elicitation, which is analogous to the clinical features of allergic asthma. HDM, house dust mite.

We firstly established an HDM-induced asthma recurrence model with reproducible clinical characteristics of asthma relapsing as re-exposed to allergen (**Figure 1A**). As shown in **Figure 1B**, after the airway resistance of model mice subsided to the similar level with control, re-exposure to HDM prominently augmented AHR evidenced by airway resistance much higher than mice being elicited primarily under the stimulation of methacholine. Consistently, after secondary elicitation, IgE level in serum, together with IL-5 and IL-13 production either in lung homogenates or BALF all significantly increased compared with that after the first elicitation (**Figures 1C, F, G**). Similar trend was confirmed in peripheral eosinophil and total cell counting in BALF as shown in **Figures 1D, E**. Furthermore, inflammatory infiltration and bronchial wall thickening was much more severe in the relapse phase as observed in **Figure 1H**.

### Administration of YPFS in Remission Phase Remarkably Attenuated Asthma Recurrence

**Figure 2:**
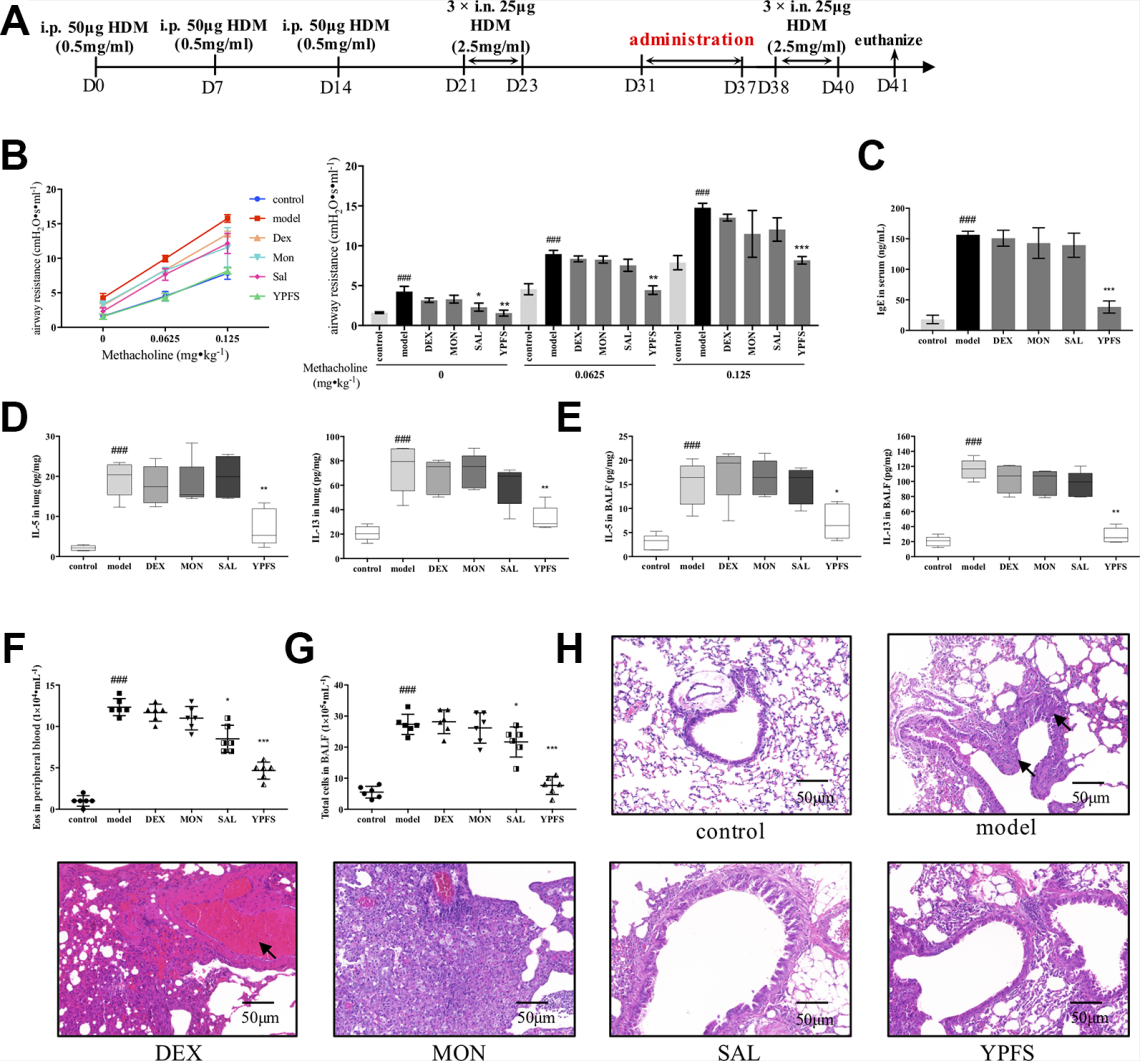
Administration of YPFS, but not three medications used in clinical, during remission showed potent efficiency against asthma recurrence. YPFS, and three medications dexamethasone (DEX), montelukast (MON) and salbutamol (SAL) were administered once per day from day 31 to day 37. **(A)** dosage regimen of YPFS, DEX, MON, and SAL on the HDM-induced asthma recurrence model; 24 h after last elicitation, **(B)** AHR of mice was evaluated. The line chart indicated that AHR of model mice escalated under stimulation of increased methacholine while YPFS significantly downregulated it. And the bar chart aside displayed statistical significance of airway resistance under stimulation of each concentration of methacholine; **(C)** IgE in serum was detected by ELISA; type 2 cytokines IL-5 and IL-13 in **(D)** lung homogenates and **(E)** BALF were detected; **(F)** Eosinophils in peripheral blood and **(G)** total cells in BALF were enumerated. (All data represent mean ± SD from three independent experiments performed in triplicate, n = 6, ^*^ *P* < 0.05, **P < 0.01, ***P < 0.001 *versus* model; ^###^ *P* < 0.001 *versus c*ontrol); **(H)** Histological changes of lung sections were determined by H&E staining (n = 6; scale bar = 50 μm). As indicated by arrows, DEX administered for 7 d induced massive pulmonary hemorrhage while MON resulted in significant inflammatory infiltration. Moreover, dosage of YPFS during remission markedly alleviated asthmatic changes in contrast with SAL. YPFS, Yu-Ping-Feng-San.

Based on the well-established HDM-induced asthma recurrence model, we firstly compared the efficacy of YPFS with three medications including corticosteroid dexamethasone (DEX), leukotriene receptor antagonist montelukast (MON) and β_2_ agonist salbutamol (SAL). All medications were all administered in remission phase (**Figure 2A**). Administered in remission phase, the effects of YPFS against asthma recurrence was determined by significantly reduced airway resistance, IgE level in serum, IL-5 and IL-13 production, peripheral eosinophils, and total cells in BALF, as well as remarkably alleviated pulmonary inflammation (**Figures 2B–H**). In contrast with YPFS, three clinically used medications administered in remission for 7 days showed no evident anti-recurrence efficiency. In addition, DEX induced abnormal weight loss (data not shown) and hemorrhage as shown in H&E staining, which are the typical adverse reactions of corticosteroid.

**Figure 3:**
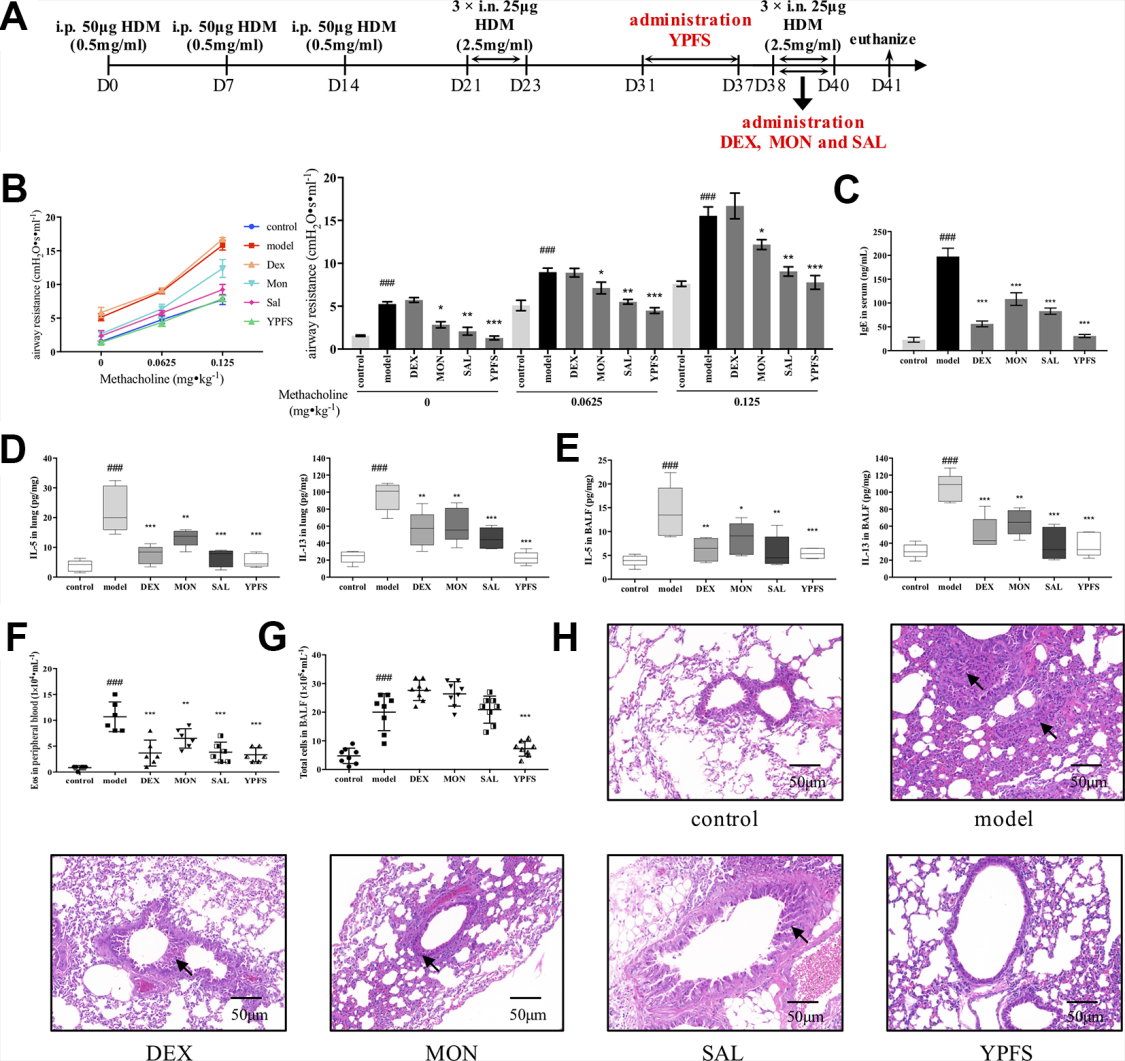
Administration of YPFS during remission period exerted more potent efficiency than three medications used during second elicitation against asthma recurrence. Since DEX, MON and SAL are commonly used when asthma exacerbates, we next administered YPFS and three medications DEX, MON, and SAL in remission and recurrence phases, respectively, to compare their efficacy against asthma recurrence. **(A)** dosage regimen of YPFS, DEX, MON, and SAL on the HDM-induced asthma recurrence model; 24 h after last elicitation, **(B)** AHR of mice was evaluated by measuring airway resistance to methacholine. The bar chart portrayed statistical analysis of airway resistance data under stimulation of each concentration of methacholine; **(C)** IgE in serum was detected by ELISA; IL-5 and IL-13 in **(D)** lung homogenates and **(E)** BALF were detected; **(F)** Eosinophils in peripheral blood, and **(G)** total cells in BALF were counted. (All data represent mean ± SD from three independent experiments performed in triplicate, n = 8, ^*^ *P* < 0.05, **P < 0.01, ***P < 0.001 *versus* model; ^###^ *P* < 0.001 *versus* control); **(H)** Histological changes of lung sections were determined by H&E staining (n = 8; scale bar = 50 μm). Black solid arrows indicated that DEX, MON and SAL alleviated inflammatory infiltration but showed negligible efficacy against bronchial wall thickening. In contrast, YPFS substantially diminished both inflammatory infiltration and thickened bronchial wall. YPFS, Yu-Ping-Feng-San; DEX, dexamethasone; MON, montelukast; SAL, salbutamol; BALF, bronchoalveolar lavage fluid.

To gain further insight into the efficacy of YPFS, we revised the dosage regimen (**Figure 3A**). YPFS was administered in remission phase while three commonly used medications were applied afterward the secondary elicitation, i.e. recurrence phase, which consistent with the clinical regimen. As shown in **Figure 3B**, all medications except DEX attenuated AHR to a certain degree. Specifically, statistical results showed that MON and SAL could reduce airway resistance compared with model, but not as potent as YPFS being capable to reduce the airway resistance to a similar level with control. Furthermore, DEX, MON, and SAL used in recurrence phase, and remission administration of YPFS all significantly decreased serum IgE level and type 2 cytokines IL-5 and IL-13 production either in lung homogenates or in BALF (**Figures 3C–E**). Cell counting results suggested all medications reduced eosinophils in peripheral blood (**Figure 3F**). YPFS administered in remission also significantly decreased total cells in BALF in contrast with that in model, while three commonly used medications applied in recurrence phase did not exert similar efficiency (**Figure 3G**). And as portrayed in **Figure 3H**, DEX administered in recurrence phase did not induce pulmonary hemorrhage as observed in **Figure 2H**. Inflammatory infiltration of lung lobes was diminished in all treatments. Moreover, YPFS remarkably attenuated bronchial wall thickening and goblet cell hyperplasia, which was more potent compared with DEX, MON and SAL.

Collectively, efficiency comparison suggested that YPFS administered in remission phase could prominently inhibit asthmatic responses in response to re-exposure of HDM, while three conventional medications used in remission or recurrence phase showed comparatively feeble efficiency with adverse reactions simultaneously.

### DSG1 Deficiency Serves as an Important Hub in Asthmatic Recurrence Model

**Figure 4:**
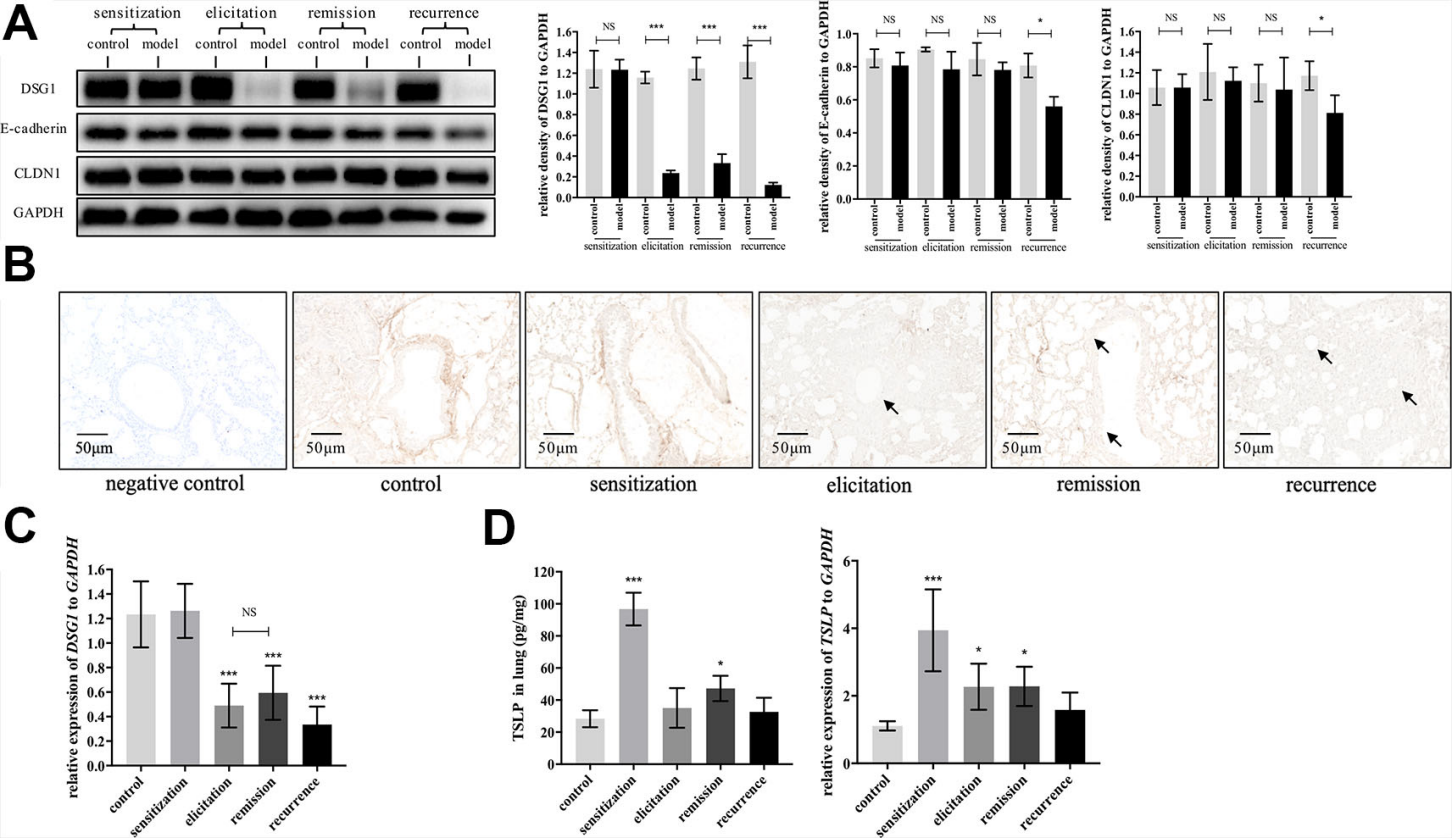
DSG1 remained deficient constantly in the asthmatic recurrence model. Dynamic changes of DSG1 and TSLP in different phases of the HDM-induced asthma recurrence model, including sensitization (day 15), elicitation (day 24), remission (day 31), and recurrence (day 41), were observed. DSG1 protein expression in different phases was determined by **(A)** Western blot and **(B)** IHC-P (n = 6, scale bar = 50 μm). Arrows pointed significantly reduced DSG1 protein around the bronchial wall after asthma exacerbated, and most importantly, DSG1 remained deficient in remission phase. Data on protein level suggested that DSG1, instead of CLDN1 or E-cadherin, might be the vital factor contributed to the compromise of bronchial epithelial barrier; **(C)** pulmonary gene expression of *DSG1* detected by RT-QPCR showed a similar trend as protein expression described above; **(D)** Protein and mRNA level of TSLP in lung in different phases of asthma recurrence model were determined by ELISA and RT-QPCR, respectively. Results indicated that pulmonary TSLP were highly expressed in remission, which was diametrically opposed to DSG1. (All data represent mean ± SD from three independent experiments performed in triplicate, n = 6, ^*^ *P* < 0.05, ***P < 0.001 *versus* control). HDM, house dust mite; DSG1, desmoglein 1; TSLP, thymic stromal lymphopoietin; NS, P ≥ 0.05.

We next evaluated dynamic changes of DSG1, E-cadherin and CLDN1, which are the main junction proteins of desmosome, AJ and TJ, respectively, in the airway during different phases of asthma recurrence model. As represented in **Figure 4A**, E-cadherin and CLDN1 only decreased after the second elicitation. In contrast, DSG1 remarkably reduced after first elicitation, and further decayed to almost negligible in recurrence phase. Most importantly, DSG1 did not restore in remission phase (**Figures 4A–C**), indicating that DSG1 degradation might play a pivotal role in the pathogenesis of asthma recurrence. Moreover, TSLP, as a key pro-allergic cytokine as reported in EoE and AD, remained higher expression in remission phase of asthma recurrence compared with control (**Figure 4D**). These data suggested that DSG1 degradation might lead to the over expression of TSLP, which contributed to the sensitive microenvironment of airway, predisposing to another asthmatic strike.

### DSG1 Is Indispensable for Bronchial Barrier Integrity

**Figure 5:**
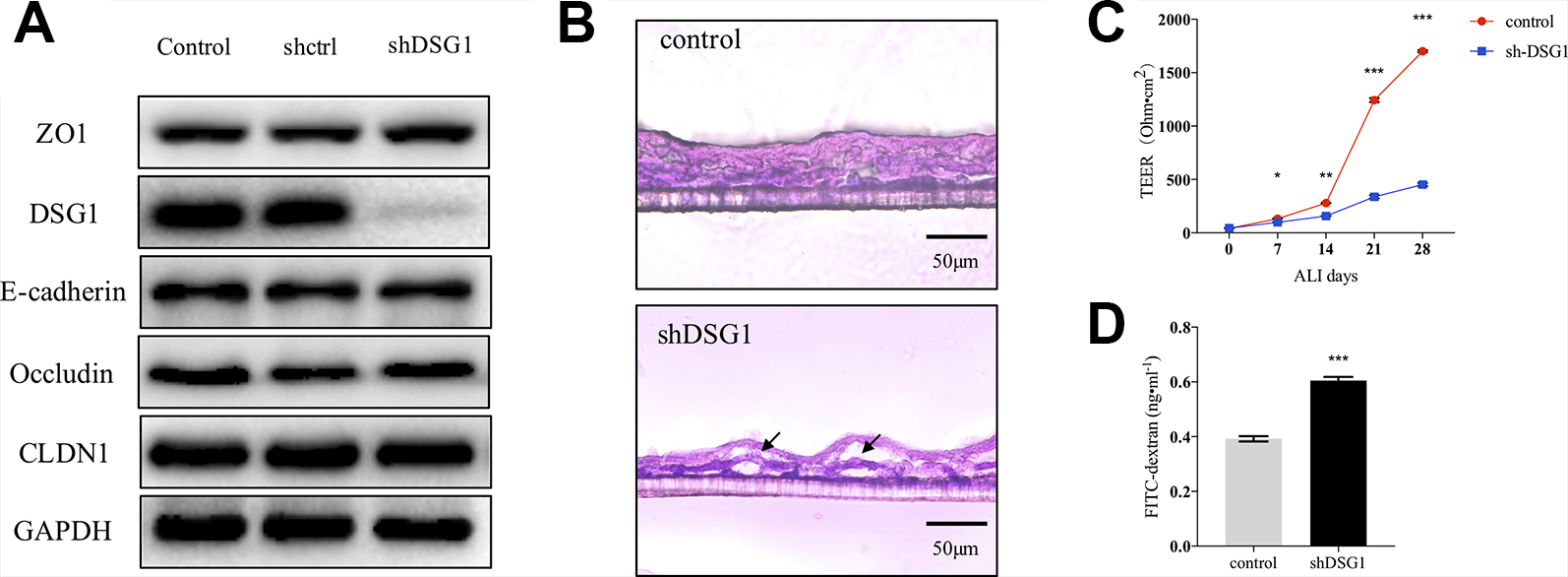
Bronchial epithelial barrier depends on DSG1. 16HBE cells (5×10^4^ per well) were seeded on Transwell^®^ permeable supports and transfected by shDSG1 after 24 h. After a confluent monolayer was formed, culture medium in the upper chamber was removed and cells were differentiated at air-liquid interface for 28 days. **(A)** shDSG1 significantly reduced the protein expression of DSG1, but displayed negligible effects on other junction proteins; **(B)** the polyester membrane of inserts was fixed in 4% paraformaldehyde and embedded in tissue freezing medium. Subsequently, sections at 6 μm were prepared and H&E staining of stratified cells was utilized to observe the effect of DSG1 knockdown (scale bar = 50 μm); Besides, the influence of shDSG1 on epithelial barrier integrity was also evaluated by **(C)** TEER measurements on ALI day 0, 7, 14, 21, and 28; and **(D)** 4kD FITC-dextran influx on day 28. Collectively, epithelial barrier was prominently compromised after DSG1 knockdown as evidenced by observable cellular separation, markedly reduced TEER and hyperpermeability of macromolecule. (All data represent mean ± SD from three independent experiments performed in triplicate, n = 3, ^*^ *P* < 0.05, **P < 0.01, ***P < 0.001 *versus* control). HDM, house dust mite; DSG1, desmoglein 1; TEER, transepithelial electrical resistance.

As described previously, DSG1 remained deficient in remission phase of asthma. We further investigated the role of DSG1 in maintaining bronchial barrier integrity utilizing short hairpin RNA and a modified ALI culture system, analogous to cell growth *in vivo*. The knockdown efficiency of shDSG1 was confirmed by Immunol blotting (**Figure 5A**). After being exposed to the ALI for 28 days, the confluent monolayers of 16HBE cells differentiated and formed a stratified bronchial epithelium (**Figure 5B**). In contrast, the silence of DSG1 prominently induced cellular separation, and epithelial barrier compromise as evidenced by significantly decreased TEER and increased 4kDa FITC-dextran flux (**Figures 5B–D**).

### HDM Treatment Induced DSG1 Compromise to Disrupt Bronchial Barrier *In Vitro*

**Figure 6:**
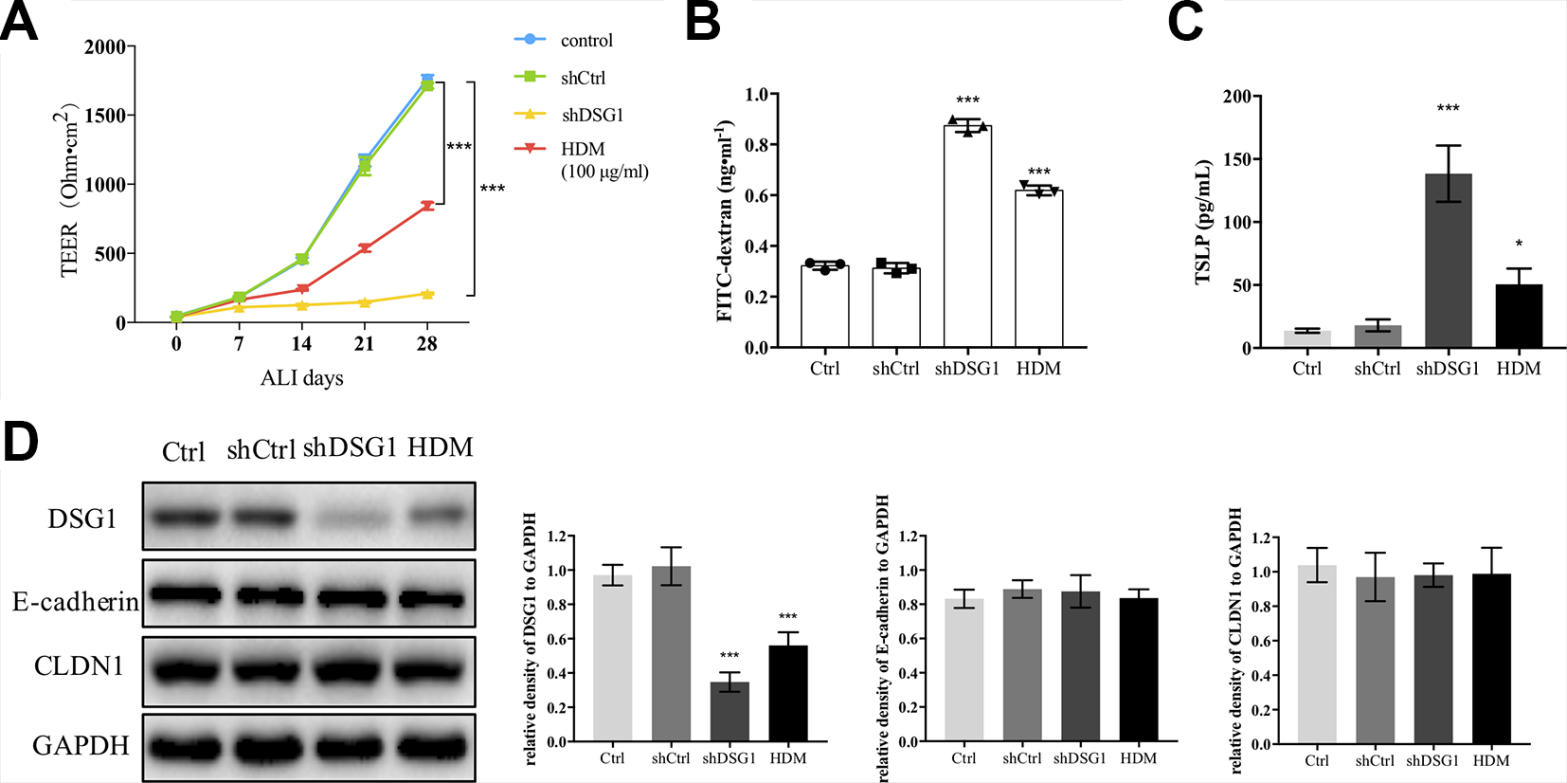
HDM-induced DSG1 deficiency resulted in epithelial barrier compromise. 16HBE cells were transfected by shDSG1 or treated with HDM at 100 μg/ml for 24 h. After forming a confluent monolayer, cells were subsequently cultured at ALI. Similar to shDSG1, HDM significantly perturbed barrier integrity as demonstrated by **(A)** reduced TEER and **(B)** escalated FITC-dextran influx; Furthermore, **(C)** TSLP in culture medium detected by ELISA and **(D)** protein expression of DSG1 determined by Western blot suggested that HDM might influence the epithelial barrier by down-regulating DSG1 and increasing the expression of TSLP. (All data represent mean ± SD from three independent experiments performed in triplicate, n = 3, ^*^ *P* < 0.05, ***P < 0.001 *versus* control). HDM, house dust mite; DSG1, desmoglein 1; TEER, transepithelial electrical resistance; TSLP, thymic stromal lymphopoietin.

Since exposure to HDM induced irrecoverable deficiency of DSG1 in the asthma recurrence model, we further confirmed the effect of HDM on DSG1 *in vitro* (**Figure 6**). After 16HBE cells formed a confluent monolayer, both treatment of 100 μg/ml HDM for 24 h and knockdown of DSG1 significantly disrupted epithelial barrier during the subsequent 28-day ALI culture demonstrated by markedly decreased TEER and elevated FITC-dextran flux (**Figures 6A, B**). Meanwhile, HDM treatment pre-ALI, consistent with the effects of shDSG1, remarkably increased TSLP in culture medium, and reduced DSG1 expression in cell lysates of ALI culture (**Figures 6C, D**). Notably, HDM showed negligible effect on E-cadherin and CLDN1, which coincided with the results *in vivo*.

### Administration of YPFS Restores DSG1 *In Vivo* and *In Vitro*

**Figure 7:**
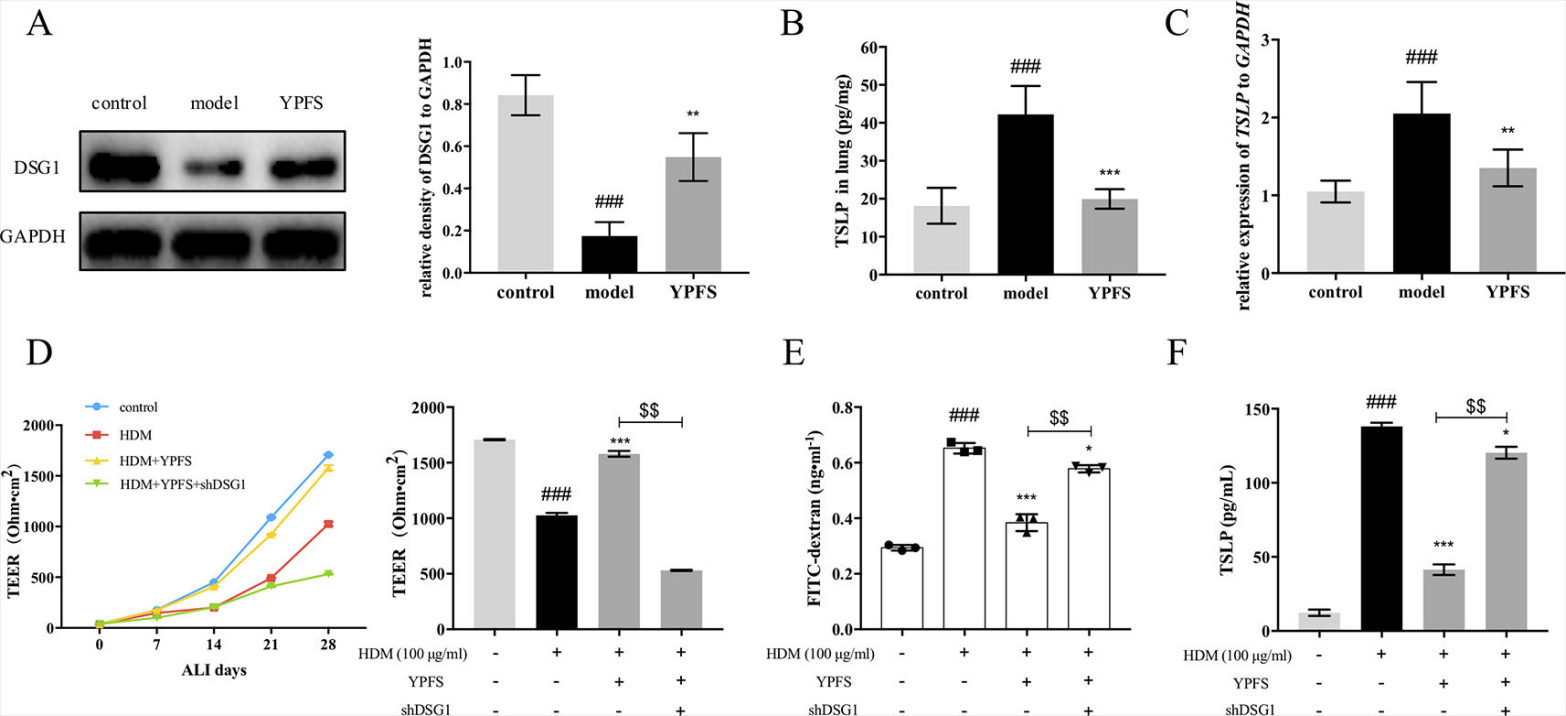
YPFS could significantly restore DSG1 deficiency and reduced TSLP *in vivo* and *in vitro*. Firstly, the effects of YPFS on DSG1 and TSLP were evaluated in the HDM-induced asthma recurrence model. **(A)** DSG1 expression in lung was detected by Western blot; Protein level **(B)** and mRNA level **(C)** of TSLP in lung was detected by ELISA and RT-QPCR, respectively. YPFS administration during remission phase was potent restoring DSG1 and degrading TSLP *in vivo*. (All data represent mean ± SD from three independent experiments performed in triplicate, n = 6, ^*^ *P* < 0.05, ***P < 0.001 *versus* model; ^###^ *P* < 0.001 *versus* control); And *in vitro*, 16HBE cells were treated with HDM at 100 μg/ml for 24 h, and then incubated with YPFS for 72 h. DSG1 were knockdown by shRNA thereafter. After forming a confluent monolayer, cells in all wells were differentiated at ALI for 28 days. Results indicated that YPFS treatment *in vitro* markedly recovered the barrier integrity as evidenced by **(D)** TEER, and **(E)** FITC-dextran influx measurement detection on day 28, which were abrogated by subsequent knockdown of DSG1; In addition, **(F)** TSLP in culture medium detected by ELISA also suggested that YPFS administration could significantly reduce the TSLP overexpression induced by HDM. However, shDSG1 afterwards remarkably elevated the TSLP expression, which suggested that YPFS might exert its effects on barrier integrity *via* DSG1 and TSLP. (All data represent mean ± SD from three independent experiments performed in triplicate, n = 3, ^###^ *P* < 0.001 *versus* control; ^*^ *P* < 0.05, **P < 0.01 *versus* HDM, ***P < 0.001 *versus* HDM; ^$$^ *P* < 0.01 *versus* HDM+YPFS). YPFS, Yu-Ping-Feng-San; TEER, transepithelial electrical resistance; TSLP, thymic stromal lymphopoietin.

We further evaluated the effect of YPFS on DSG1 both in the HDM-induced asthma recurrence model and the ALI culture of 16HBE cells. As shown in **Figure 7A**, DSG1 reduced in remission phase whereas YPFS remarkably restored this desmosomal cadherin. Moreover, YPFS significantly reduced pulmonary TSLP production which significantly increased during the remission phase of the asthma recurrence model (**Figures 7B, C**).

*In vitro*, 100 μg/ml HDM pre-treatment on 16HBE reduced TEER and increased FITC-dextran flux, indicating the compromise of the stratified bronchial barrier after ALI culture (**Figures 7D, E**). In contrast, YPFS prominently restored TEER and drew down the flux of FITC-dextran. More importantly, subsequent silence of DSG1 after administration of YPFS abolished the effect of both treatments, suggesting that YPFS was capable to restore barrier integrity through up-regulating DSG1. Furthermore, the TSLP inhibiting capability of YPFS was reversed by DSG1 silence, as a consequent of bronchial barrier restoration (**Figure 7F**).

## Discussion

As one of the most refractory chronic disease, asthma has caused considerable public health problems globally. Urgently, clinicians and researchers should promote novel approaches to tackle this refractory disease. In this regard, the Chinese prescription YPFS applied for reducing allergic responses like AD recurrence, etc., also exerts potent capability of inhibiting asthma recurrence clinically. Of particular note is that YPFS is commonly used during the remission phase of asthma, in contrast with three conventional medications employed in acute exacerbation.

To investigate the underlying mechanisms of YPFS against asthma, we duplicated an optimized asthma recurrence model. OVA and HDM are two major allergens used to establish experimental allergic asthma model (Maltby et al., 2017). Firstly, by comparing the asthmatic responses of an OVA and DBP-challenged model and three different HDM-induced models, we screened out the optimal mice model. The 24-day-long regimen duplicated with HDM remarkably increased AHR, and augmented type 2 inflammatory responses in airway. Subsequently, we established an HDM-induced asthma recurrence model by re-challenge after AHR of model mice recovered to normal level, which was analogous to the clinical features of asthma relapse in human subjects. As expected, second elicitation prominently augmented the hallmark features of allergic asthma which were previously observed in the original HDM-induced asthma model. In hence, the asthma recurrence mice model we established provided a basic tool which not only helped us to determine the key changes during remission, but also to elucidate the mechanisms of YPFS against asthma recurrence.

Utilizing the HDM-induced asthma recurrence model, we further evaluated the potency of YPFS against asthma relapse. Based on the HDM-induced asthma recurrence model, we compared the efficiency of YPFS administered in remission versus three medications applied in clinical including dexamethasone, salbutamol and montelukast. Since lung inflammation is the significant cause of asthma, anti-inflammation corticosteroids are the most commonly used medications to control the attack of asthma (He et al., 2019). In particular, β_2_ agonists like salbutamol and (or) leukotriene receptor antagonists represented by montelukast might be used as the combination of corticosteroids to control moderate-to-severe asthma (Pedersen and O'Byrne, 2012; Bateman et al., 2016). However, the clinical application of these agents still encounters three main problems or obstacles. First of all, patients with severe asthma may have steroid resistance (He et al., 2019). Second, these medications are developed to control asthma symptoms instead of curing the disease. Third, long-term use of these agents causes a variety of adverse effects (Dempsey, 2000; Tse et al., 2012; Patel et al., 2014). Our results indicated that the three conventionally used medications administered in remission phase did not show observable effects against asthma recurrence. Indeed, the corticosteroid dexamethasone used for 7 days during remission led to pulmonary hemorrhage and significant body weight loss of model mice. In sharp contrast, administration of YPFS in remission exerted remarkable inhibitory efficacy against asthma recurrence, which was even more potent compared with the effects of those widely applied medications administered during second elicitation. Meanwhile, YPFS administration showed negligible side effects, consisting with the common advantages of TCM.

Bronchial epithelial barrier dysfunction is etiologically involved in asthma (Georas and Rezaee, 2014). As reported previously, DSG1 dysfunction results in severe skin dermatitis, multiple allergies and metabolic wasting, i.e. the SAM syndrome, indicating that this cadherin plays a key role in maintaining epidermal barrier (Samuelov et al., 2013). Besides, the deficiency of DSG1 induced by IL-13 might weaken esophageal epithelial integrity, increase periostin and epithelium-secreted TSLP and potentiate EoE, an inflammatory disorder at mucosal epithelium (Sherrill et al., 2014; Davis et al., 2016). In addition, recent data showed that DSG1 also expressed in oral mucosa (Kumar et al., 2017), and most importantly, in human bronchial epithelial cells (HBEC) (Saaber et al., 2015). Considering DSG1-associated epithelial barrier dysfunction is pivotal in skin and mucosal inflammatory diseases, it is highly possible that the abnormal expression of this desmosomal cadherin at bronchial epithelium might also contribute to the pathogenesis of chronic asthma. To verify this hypothesis, we observed the dynamic changes of CLDN1, E-cadherin and DSG1 in lung tissue in different phases of asthma recurrence model, especially during the remission period. Of note, DSG1 significantly reduced after HDM elicitation and remained deficient compared with that in control after AHR recovered to normal level, while CLDN1 and E-cadherin only decreased after second elicitation. Therefore, the abnormal expression of DSG1 might be the key factor resulting in damaged bronchial barrier.

To further verify the significance of DSG1 in maintaining bronchial barrier, ALI culture of human bronchial epithelial cell line 16HBE was employed. Silence of DSG1 did not affect the expression of a variety of TJs and AJs, but dramatically induced cellular separation and disrupted barrier integrity of the well-differentiated stratified epithelium *in vitro*. By this we confirmed that DSG1 was indispensable for bronchial epithelial barrier. More importantly, HDM treatment pre-ALI culture also reduced DSG1, but not CLDN1 and E-cadherin, and subsequently led to relatively moderate but significant compromise of epithelial barrier. Collectively, DSG1 contributed crucially to bronchial barrier integrity, and HDM could induce long-term deficiency of DSG1 and abnormal expression of TSLP which might result in a sensitive microenvironment at bronchial epithelium, and lead to asthma relapse upon another allergen exposure.

Finally, the correlation between the potency of YPFS against HDM-asthma relapse and DSG1 was evaluated *in vivo* and *in vitro*. Data showed that YPFS could significantly upregulate the expression of DSG1 and inhibit the overexpression of TSLP in remission phase of the asthma recurrence model. Most intriguingly, YPFS treatment restored epithelial barrier integrity of ALI-cultured 16HBE and reduced TSLP in culture medium, which was significantly abrogated by subsequent DSG1 knockdown. These data suggested that it was by regulating DSG1 that YPFS possessed its protective efficiency on bronchial epithelial barrier.

Collectively, the dysfunction of DSG1 during the remission phase might contribute to bronchial barrier compromise and overexpression of TSLP, which facilitated asthma relapse in a faster and more severe pattern. Furthermore, YPFS could significantly inhibit asthma recurrence by restoring abnormal decreased DSG1 during remission period. Inspiringly, our results provide a novel and potent therapeutic approach against asthma recurrence. Or at least, the combined application of YPFS in remission with agents like inhaled corticosteroid during attack might be an optimal solution of chronic severe asthma.

## Data Availability Statement

The datasets generated for this study are available on request to the corresponding author.

## Ethics Statement

The animal study was reviewed and approved by the Animal Care and Use Committee of Nanjing University of Chinese Medicine. Written informed consent was obtained from the owners for the participation of their animals in this study.

## Author Contributions

KB and MH designed the experiments. KB, YJZ, YC, WY, XRY, XYW, ZJ, XY, XTW, LY, SW, YX, and YHZ performed the experiments and collected data. The data analysis was conducted by KB, JZ, and MH. And the manuscript was written, revised, and edited by KB and MH.

## Funding

We appreciate the funding from the National Natural Science Foundation of China (grant numbers 81473395 and 81473390); the Priority Academic Program Development of Jiangsu Higher Education Institutions (PAPD); the Natural Science Foundation of Jiangsu Province (grant number BK20141466); and Jiangsu Key Laboratory for Pharmacology and Safety Evaluation of Chinese Materia Medica (grant number JKLPSE201603).

## Conflict of Interest

Author XY was employed by The Nanjing Han & Zaenker Cancer Institute (NHZCI), OG Pharmaceuticals.

The remaining authors declare that the research was conducted in the absence of any commercial or financial relationships that could be construed as a potential conflict of interest.

## Supplementary Material

The Supplementary Material for this article can be found online at: https://www.frontiersin.org/articles/10.3389/fphar.2019.01698/full#supplementary-material
