# Supplementary material for: A Chinese Prescription Yu-Ping-Feng-San Administered in Remission Restores Bronchial Epithelial Barrier to Inhibit House Dust Mite-Induced Asthma Recurrence
Source: Front Pharmacol. 2020 Jan 31;10:1698. doi: 10.3389/fphar.2019.01698 (PMC7006455; doi:10.3389/fphar.2019.01698)
Supplement: Supplementary file 1 [file DataSheet_1.pdf]

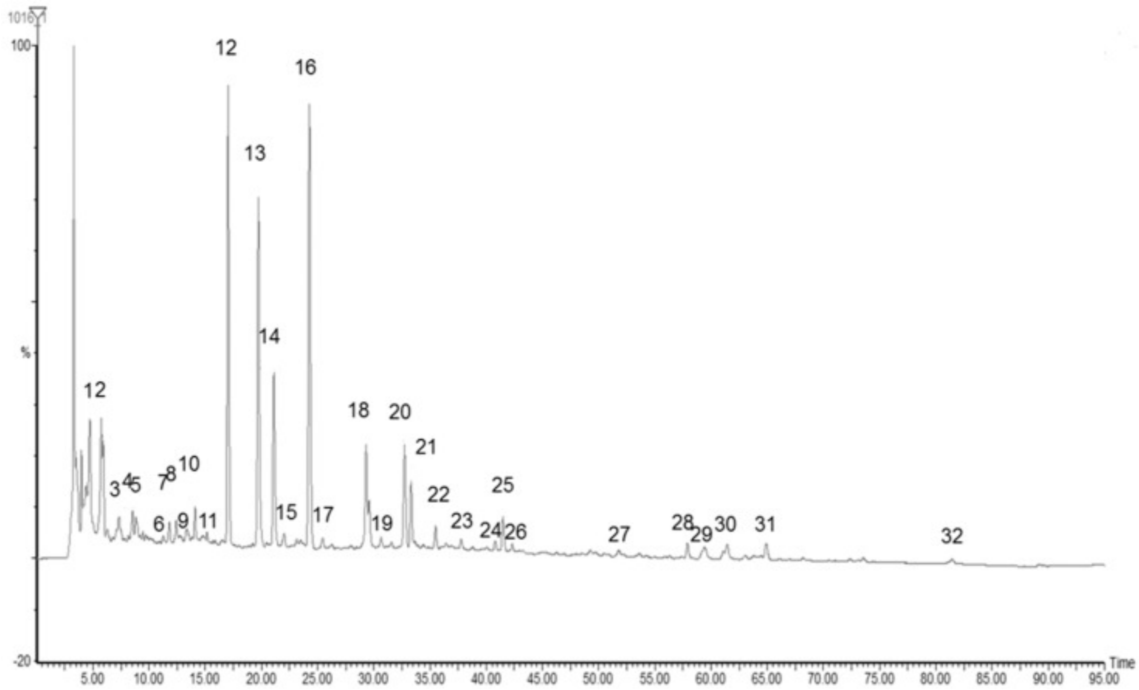

Figure S1 HPLC fingerprint of YPFS extract at 254 nm. A total of 32 main peaks were detected in the fingerprint of YPFS established by HPLC-MS (doi: 10.1371/journal.pone.0107279).

Table S1 Relative retention time (RRT) and relative peak area (RPA) of each peak in the fingerprint of YPFS extract (doi: 10.1371/journal.pone.0107279).

No.	RRT	RPA	Identified compounds
1	0.195039	0.409416	
2	0.237648	0.4078	
3	0.301521	0.077928	
4	0.35163	0.056677	
5	0.363823	0.0987593	
6	0.463535	0.0121612	
7	0.486958	0.039583	
8	0.511683	0.041818	
9	0.54162	0.037255	
10	0.581036	0.047355	
11	0.625051	0.031994	
12	0.7019	0.843082	Prim-O-glucosylcimifugin
13	0.813162	0.766692	Calycosin-7-glucoside copyranoside
14	0.869494	0.394774	Cimifugin
15	0.906581	0.035079	
16	1	1	4′-O-glucopyranosyl-5-O-methylvisamminol
17	1.048749	0.02977	
18	1.208102	0.367158	Ononin
19	1.263032	0.025007	

20	1.348869	0.247059	Calycosin
21	1.372934	0.129639	Sec-o-glucosylhamaudol
22	1.462892	0.047043	
23	1.55697	0.021852	
24	1.681296	0.020301	
25	1.710141	0.082697	Formononetin
26	1.743808	0.018922	
27	2.134322	0.017997	
28	2.386657	0.038159	
29	2.448469	0.067835	
30	2.531545	0.056232	
31	2.675114	0.04355	
32	3.351017	0.02101	
